# Intra-Section Analysis of Human Coronary Arteries Reveals a Potential Role for Micro-Calcifications in Macrophage Recruitment in the Early Stage of Atherosclerosis

**DOI:** 10.1371/journal.pone.0142335

**Published:** 2015-11-10

**Authors:** Martijn L. L. Chatrou, Jack P. Cleutjens, Ger J. van der Vusse, Ruben B. Roijers, Peter H. A. Mutsaers, Leon J. Schurgers

**Affiliations:** 1 Department of Biochemistry, Cardiovascular Research Institute Maastricht, Maastricht University, Maastricht, the Netherlands; 2 Department of Pathology, Cardiovascular Research Institute Maastricht, Maastricht University, Maastricht, the Netherlands; 3 Department of Physiology, Cardiovascular Research Institute Maastricht, Maastricht University, Maastricht, the Netherlands; 4 Cyclotron Laboratory, Department of Applied Physics, Eindhoven University of Technology, Eindhoven, the Netherlands; INSERM, FRANCE

## Abstract

**Background:**

Vascular calcification is associated with poor cardiovascular outcome. Histochemical analysis of calcification and the expression of proteins involved in mineralization are usually based on whole section analysis, thereby often ignoring regional differences in atherosclerotic lesions. At present, limited information is available about factors involved in the initiation and progression of atherosclerosis.

**Aim of This Study:**

This study investigates the intra-section association of micro-calcifications with markers for atherosclerosis in randomly chosen section areas of human coronary arteries. Moreover, the possible causal relationship between calcifying vascular smooth muscle cells and inflammation was explored *in vitro*.

**Technical Approach:**

To gain insights into the pathogenesis of atherosclerosis, we performed analysis of the distribution of micro-calcifications using a 3-MeV proton microbeam. Additionally, we performed systematic analyses of 30 to 40 regions of 12 coronary sections obtained from 6 patients including histology and immuno-histochemistry. Section areas were classified according to CD68 positivity. *In vitro* experiments using human vascular smooth muscle cells (hVSMCs) were performed to evaluate causal relationships between calcification and inflammation.

**Results:**

From each section multiple areas were randomly chosen and subsequently analyzed. Depositions of calcium crystals at the micrometer scale were already observed in areas with early pre-atheroma type I lesions. Micro-calcifications were initiated at the elastica interna concomitantly with upregulation of the uncarboxylated form of matrix Gla-protein (ucMGP). Both the amount of calcium crystals and ucMGP staining increased from type I to IV atherosclerotic lesions. Osteochondrogenic markers BMP-2 and osteocalcin were only significantly increased in type IV atheroma lesions, and at this stage correlated with the degree of calcification. From atheroma area type III onwards a considerable number of CD68 positive cells were observed in combination with calcification, suggesting a pro-inflammatory effect of micro-calcifications. *In vitro*, invasion assays revealed chemoattractant properties of cell-culture medium of calcifying vascular smooth muscle cells towards THP-1 cells, which implies pro-inflammatory effect of calcium deposits. Additionally, calcifying hVSMCs revealed a pro-inflammatory profile as compared to non-calcifying hVSMCs.

**Conclusion:**

Our data indicate that calcification of VSMCs is one of the earliest events in the genesis of atherosclerosis, which strongly correlates with ucMGP staining. Our findings suggest that loss of calcification inhibitors and/or failure of inhibitory capacity is causative for the early precipitation of calcium, with concomitant increased inflammation followed by osteochondrogenic transdifferentiation of VSMCs.

## Introduction

Vascular calcification (VC) is clinically related to poor diagnosis and has been independently associated with increased cardiovascular mortality and morbidity. The amount of calcification, as measured and quantified by multi-slice computed tomography (MSCT), is being used as a marker for atherosclerotic burden and has been proven to be an important predictor of all-cause mortality, vascular complications and myocardial infarctions [1,2]. Additionally, patients with a coronary calcification-progression of more than 15% per year have a 17-fold increased risk of suffering from myocardial infarction compared to patients without significant VC progression [1–3].

Currently, there is no consensus about the concept of the initiation and prolongation of VC. A lack of sensitive detection methods is one of the limiting factors in elucidating this issue. It is of fundamental interest to investigate the involvement of micro-calcification in atherogenesis. Among others, the presence of micro-calcifications has been shown to be detrimental for plaque stability [4]. Recently, our group and others have shown that calcification is already present at early stages of atherosclerosis [5,6]. These micro-calcifications were shown to correlate with macrophages and osteochondrogenic activity in early-stage atherosclerosis, suggesting a key-role for micro-calcification induced inflammation. This notion was supported by *in vitro* work in which calcium crystals induced a pro-inflammatory macrophage response [7] and apoptosis of VSMCs [8].

Previous studies on the processes involved in the earliest events resulting in VC were performed by immuno-histochemical inspection of human atherosclerotic lesions and associated intimal calcium depositions with plaque progression. Routinely, the severity of the atherosclerotic lesion was classified according to the AHA [9] and performed on the basis of standard staining procedures which accounts for the whole section. However, routine assessment of atherosclerotic lesions underestimates the regional heterogeneity found in atherosclerotic lesions and thereby development of atherosclerotic plaques from regional heterogeneity [10]. Moreover, an immuno-histochemical approach cannot be performed longitudinally in time. According to AHA criteria, calcification is commonly only recognized in complex and advanced atheromas. Measurement of atherosclerotic sections with a 3-MeV proton beam allows us detection of calcification at (sub) micrometer scale in early type atherosclerotic plaques. Taking advantage of the heterogeneity of the atherosclerotic lesions within one section of human coronary arteries a more detailed analysis of the processes under investigation could be performed by selection of different regions within the same section. Therefore, we used multiple chosen regions within one section and thus reduced the inter-individual variation between sections. To the best of our knowledge this approach has never been used before, and provided us with data to elucidate the initiation processes on the genesis of atherosclerosis. Moreover we performed additional *in vitro* experiments to further investigate the pro-inflammatory profile of calcifying VSMCs.

## Materials and Methods

### Elemental analysis

The elemental composition of human coronary arteries was assessed with a 3-MeV proton beam generated by a 3.5-MV accelerator (Singletron [11]. High Voltage Engineering Europe B.V., Amersfoort, the Netherlands) at the Eindhoven University of Technology, Eindhoven, the Netherlands. Element analyses were performed using Particle-Induced X-ray Emission (PIXE) in combination with backscattering and forward-scattering spectroscopy. Using PIXE analysis elements such as Ca could be identified. Detailed information of the analysis is previously described [5,12].

### Tissue preparation

Twelve coronary arteries were collected during autopsy of six patients (aged 47 to 86 years) who died from non-cardiac causes. Autopsy was performed 6 to 9 hours after death (Department of Pathology, Academic Hospital Maastricht, Maastricht). Tissue collection was approved by the Maastricht Pathology Tissue Collection committee [5]. The Medical Ethics Committee of the Maastricht University approved the study protocol and all subjects gave their informed consent in writing.

The tissue samples, placed in Tissue Tek (Sakura, Zoeterwoude, the Netherlands), were frozen in liquid nitrogen and stored at -80°C. Tissue samples were sectioned in 5 μm thick sections and collected on glass slides and stored at -20°C or collected on pre-dried Pioloform (Agar Scientific LTD, Stansted, UK) films of 100 nm for PIXE analysis.

### Immuno-histochemical Staining

Immuno-histochemistry was performed on frozen sections stained with the primary antibodies for a-SMA (clone 1A4; Dako, Glostrup, Denmark), CD68 (clone KP1, Dako), uncarboxylated and carboxylated MGP (ucMGP and cMGP, respectively; 1:25; IDS, Boldon, UK), BMP-2 (1:20; Genetics Institute, Cambridge, MA) and Osteocalcin (1:50; Anawa Trading, Wangen, Zürich, Switzerland). Secondary antibodies used were Biotinylated sheep anti-mouse IgG (1:250; Amersham, Little Chalfont, Buckinghamshire, UK) or sheep anti-rabbit IgG (1:1000, Dako). Antibodies were visualized by alkaline phosphatase–coupled avidin-biotin complex (Dako), in combination with red alkaline substrate kit I (Vector SK-5100; Vector Laboratories, Burlingame, CA); nuclei were counterstained with hematoxylin. Furthermore, all samples we routinely stained for Hematoxylin Eosin (HE), von Kossa, oil red O and Picro-Sirius red.

### Histochemical analyses

Quantitative analyses of the histochemical findings were done using in-house programmed macros for image processing software imageJ (ImageJ; US National Institutes of Health, Bethesda, MD, USA). The calcium yield scans obtained with the 3-MeV proton beam were converted to a processable image and intensities were converted to grey values. Next, grey values were quantified, resulting in a number corresponding to calcified area normalized by total intimal area.

Analysis of immuno-histochemical stained sections was determined by “Method one”—quantification of positive staining normalized on total intimal tissue surface area or “Method two”—cell specific staining by counting positive stained pixels in a defined area around nuclei (blue; Hematoxylin) and alkaline substrate (red; substrate of Vector Labs) using ImageJ colour deconvolution. In the second Method, a region of interest (ROI) was drawn around nuclei and the sum of all positive stained pixels in the ROIs was calculated. This number was normalized by the total amount of pixels covered by the ROIs. Detailed information on image analyses are described in “Supporting information”. Immuno-histochemical staining was quantified according to “Method two” except for MGP. MGP positivity was calculated according to “Method one”, since MGP is often found in non-cellular regions because of MGP binding to calcium-phosphate crystals [13,14].

### Tissue lesion analysis and classification

A selection of regions was performed in 12 samples of human atherosclerotic coronary arteries. Within the section, we selected between 3–4 regions, resulting in a total of 40 regions for further analysis. These regions were classified according to CD68 positivity. CD68 positivity (as determined according to “Method two”) below 3% was classified as type I, between 3–10% as type II, between 10–30% as type III and above 30% as type IV atherosclerotic lesions. Adjacent sections were stained for von Kossa positivity and analyzed with immuno-histochemical staining for calcification-regulating proteins. Additionally, calcium yield scans were made with the 3-MeV proton beam (beam size, 3.0 x 3.0 μm^2^) in adjacent sections [5].

### Invasion assay

To test chemoattractant activity of calcifying human primary VSMCs on macrophages, an invasion assay using the xCELLigence system (Roche/ACEA Biosciences, Mannheim, Germany) was performed. Firstly, conditioned medium was obtained by calcification of VSMC with 5.4 mM Ca^2+^ and 2.5% fetal calf serum (FCS) for 48h. Non-calcified conditioned medium was obtained from non-calcified cells on 1.8 mM Ca^2+^ and 2.5% FCS. The conditioned medium was centrifuged at 1150 x g to dispose of apoptotic bodies and other cellular debris. The calcium ion concentration of the conditioned medium was measured by o-cresolphthalein complexone method for later adjustment of calcium concentration in non-calcified conditioned medium before use in the invasion assay. This correction nullified possible influences of chemoattractant activity of extracellular calcium.

Secondly, THP-1 cells were stimulated with 50 nM phorbol 12-myristate 13-acetate (PMA; Promega, Madison, USA) for 24h to obtain macrophages. Both upper (40 μg/ml) and lower wells (10 μg/ml) were coated with collagen (collagen from bovine skin; Biochrom). Stimulated THP-1 cells (40 x 10^3^) were starved for 6h in 0.5% FCS and inserted into the upper chamber. Conditioned medium of calcified and non-calcified cells (adjusted to the same calcium concentration) was used as a chemoattractant in the lower chamber. All experiments were done in triplicate and in 2.5% FCS in both lower and upper chamber.

### cDNA synthesis and quantitative analysis

Total RNA was extracted from human primary VMSC using tri reagent (Sigma, Zwijndrecht, the Netherlands). RNA concentrations were quantified spectrophotometrically at 260 nm. RNA integrity was evaluated using denaturing agarose gel electrophoresis. 250 ng of total RNA was treated with DNase I (Promega, Leiden, the Netherlands). The purified RNA was reverse transcribed using Moloney Murine Leukemia Virus Reverse Transcriptase (M-MLV RT) for 1 h at 37°C, in the presence of RNAse Out, dNTPs, dithiothreitol (all Invitrogen, Bleiswijk, the Netherlands), and an oligo(dT) primer (Eurogentec, Maastricht, the Netherlands). Gene expression levels were quantified by real-time quantitative PCR (qPCR) in a LightCycler 480 (Roche Applied Science, Almere, the Netherlands). Amplification reactions were carried out in a volume of 10 μl, containing 100 ng of total cDNA, 5 μl QuantiTect SYBR Green PCR Kit (Qiagen) and 0.5 μM of 5’ and 3’ primers (Eurogentec). An initial denaturation step (15 min at 95°C) was followed by 50 cycles of amplification (denaturation: 15 s at 95°C, annealing: 30 s at 57°C, extension: 45 s at 72°C). Melting curve genotyping was performed to check the specificity of amplification products. Fluorescence curves were analyzed with LightCycler 480 Software (Version 1.5) and relative quantification was performed with the 2^−ΔΔCt^ method. All samples were assayed in triplicate.

### Statistics

The results are presented as mean ± SD. Statistical analysis was performed by one-way ANOVA with linear trend posthoc test by unpaired non-parametric t test (Mann-Whitney) or linear regression analysis as appropriate using PRISM software (GraphPad). Pearson’s correlation coefficient was calculated to analyze statistical correlation. Values of P < 0.05 were considered statistically significant.

## Results

### Classification of regions in coronary artery specimens

**Fig 1:**
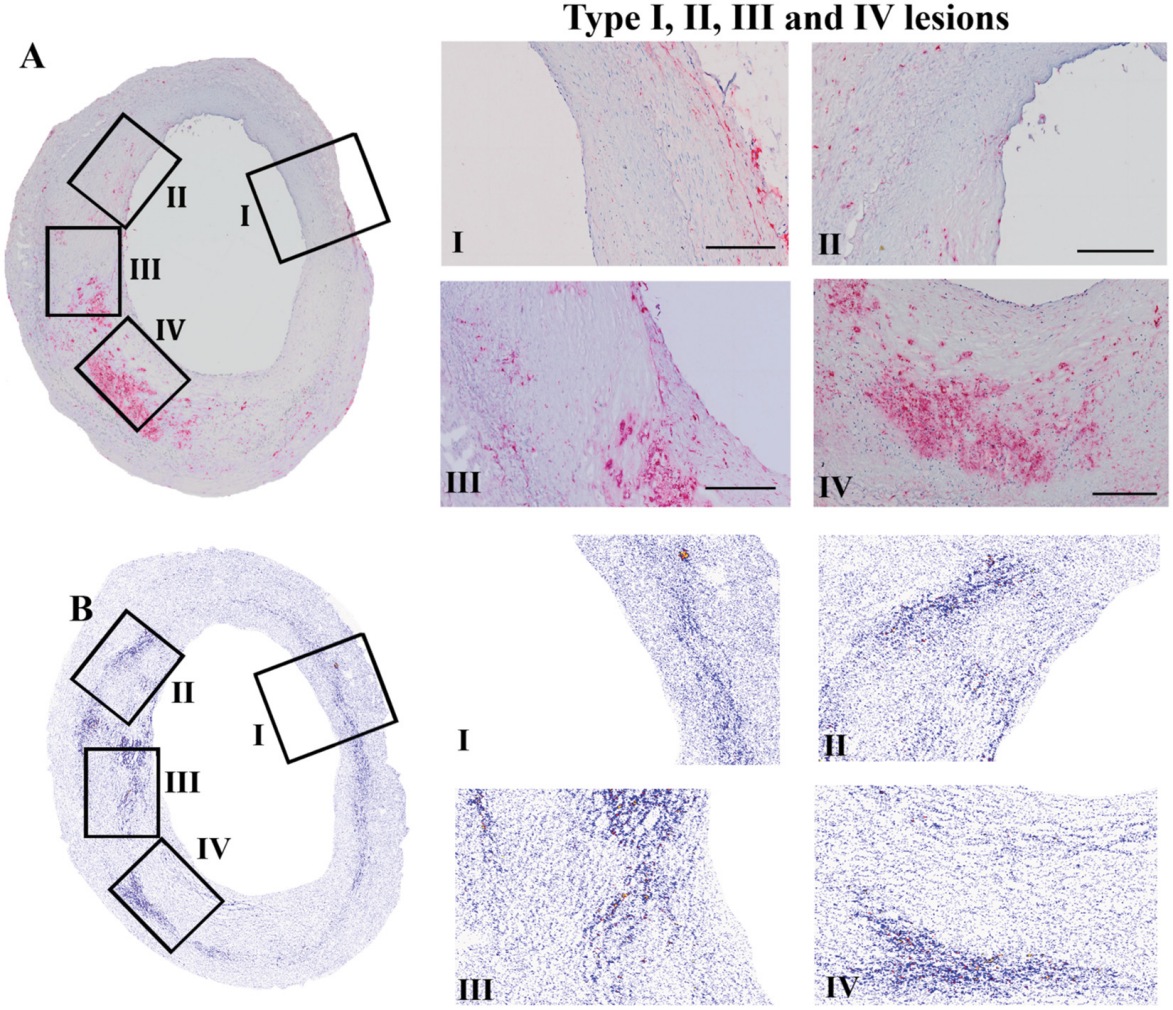
Regional differentiation of atherosclerotic lesion types. An overview of a human coronary artery section, depicting atherosclerotic stages I to IV, is shown in the left panel, with enlargements of the selected regions at the right panel. The regions of coronary lesions classified as types I, II, III, and IV, based on CD68 positivity, are shown in 1A, with corresponding regions in the calcium yield scan in 1B.

In a previous report, we used 12 samples of human coronary artery walls of six patients [5]. These sections were classified and accordingly analyzed on the basis of criteria described by Stary [9]. In the present study we took advantage of the heterogeneity within one section to perform a semi-longitudinal investigation and to decrease the effect of inter-individual variability when studying whole sections (Fig 1). All selected regions within one section were classified on basis of increased CD68 staining (Fig 1A). The threshold (as described in Materials and Methods) set for stratification based on CD68 staining corresponded well to regional classification according to AHA criteria [9] as judged by two independent experts in the field (data not shown). On the basis of CD68 staining at least 3 regions classified with different severities (type of lesions) could be selected per tissue section.

### Qualitative analysis of atherosclerotic stage-related regions of coronary artery specimens

#### Type I lesions

**Fig 2:**
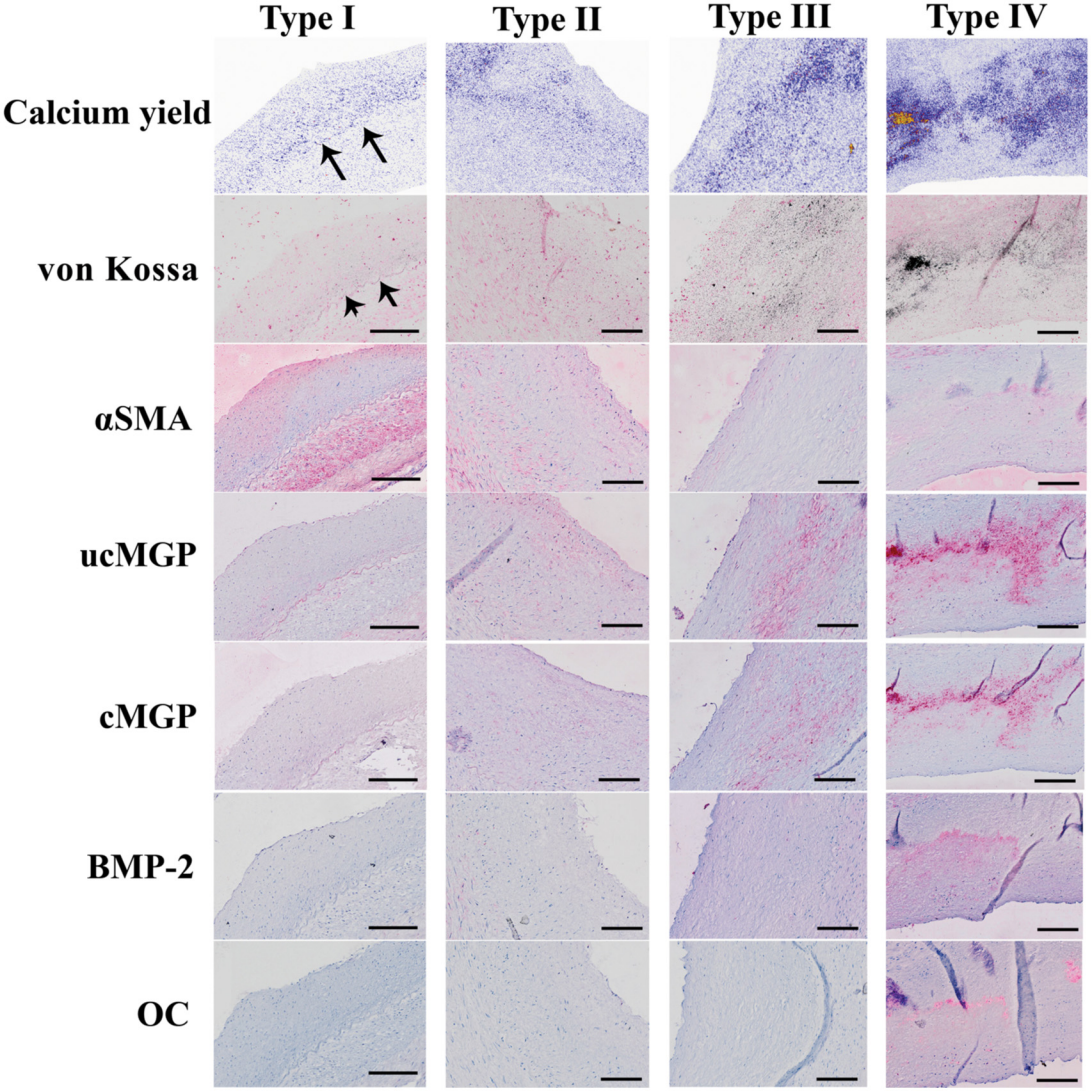
Representative images of type I, II, III and IV regions. Representative images of regions classified as type I, II, III and IV with the corresponding calcium yield scan and (immuno-)histochemical staining of von Kossa, αSMA, ucMGP, cMGP, BMP-2 and osteocalcin (OC) in adjacent sections. Arrows indicate calcification close to the internal elastic lamina. Scale bars are 200μm.

Stratification of lesions according to macrophage content revealed that already in the earliest stages of atherosclerosis micro-calcifications were present (section with type I lesion, Fig 1B). CD68 positive cell content was low in stage I lesions and stained on average 3% of total cell area (data not shown). Calcification in type I lesions was typically located close to the internal elastic lamina (indicated by arrows), and co-stained with uncarboxylated MGP (Fig 2, ucMGP stain). Furthermore, ucMGP positivity was mainly found at the elastica interna, dividing intima and media (Fig 2). Osteochondrogenic markers such as BMP-2 and osteocalcin (Fig 2, BMP-2 and osteocalcin stain) could not be detected, indicating that osteochondrogenesis is not key in the early stage of calcification or atherogenesis.

#### Type II lesions

Also in type II atherosclerotic regions micro-calcifications were abundantly present. In type II lesions calcification was more spread within the intima and progressed towards the lumen (Fig 2; calcium yield scan). VSMCs located close to the internal elastic lamina progressively lost longitudinal orientation, indicating an increased content of synthetic VSMC. Additionally, in stage II lesions osteochondrogenic markers were still absent (Fig 2; BMP-2 and osteocalcin stain).

#### Type III lesions

The progression of atherogenesis towards type III increased the amount of calcification in the affected region. Typically at this stage regions contain fewer nuclei, often accompanied by an a-cellular core. The presence of an a-cellular core with surrounding collagen-rich extracellular matrix correlates with speckled fragments of calcification and often coalesced into larger crystals (Fig 2, von Kossa stain). At this stage osteochondrogenic markers are observed in some cases.

#### Type IV lesions

Increased calcification and macrophage infiltration resulted in more osteochondrogenic positive cells (BMP-2, osteocalcin), as observed in type IV atherosclerotic regions (Fig 2, BMP-2, OC). At this stage loss of regional nuclei is progressed and amount of calcifications in the form of larger crystals is increased.

**Fig 3:**
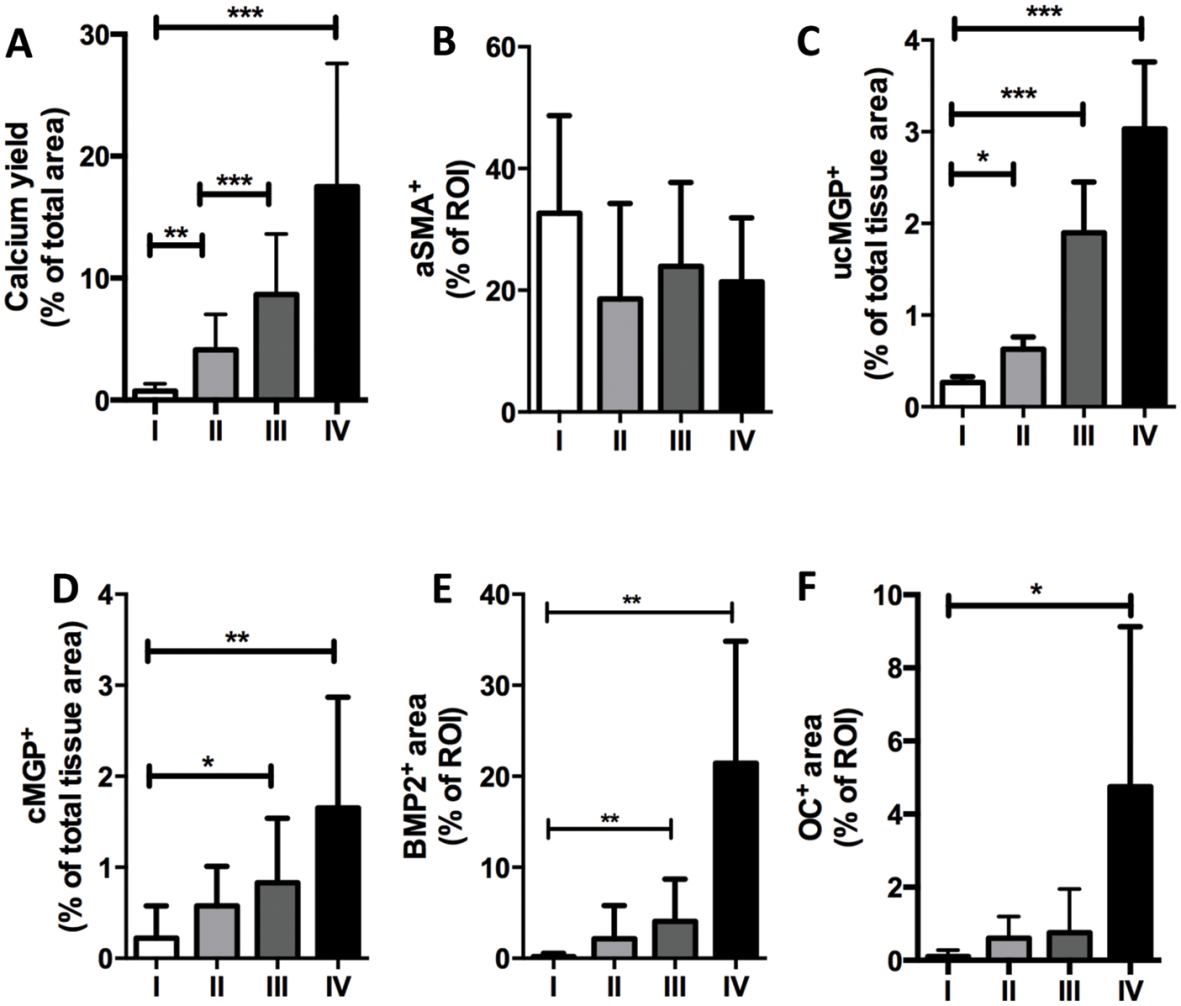
Correlation of degree of atherosclerotic lesion with micro-calcification and proteinous catalysts/inhibitors of calcification. Intimal regions of coronary lesions were chosen and classified as types I, II, III, and IV based on CD68 positivity. Calcification was calculated in corresponding regions using the Calcium yield scan (S1 File). Immuno-histochemical analysis of proteinous catalysts and inhibitors of calcification was performed according to “Method one” for CD68, aSMA, OC and BMP2 (as described in S2 File), and according to “Method two” for cMGP and ucMGP (as described in S3 File). The number of observations ranged from 6 to 8 for type I lesions and between 9 and 12 for type II, III and IV lesions. *P < 0.05, **P < 0.001, ***P < 0.0001, significance was assessed by unpaired non-parametric t test (Mann-Whitney).

Quantitative analysis revealed that the amount of micro-calcifications significantly increased from type I towards type IV lesions (Fig 3A). Only uncarboxylated MGP followed the enhancement pattern of micro-calcifications closely and significantly increased from type I lesions to type IV lesions (Fig 3C). Osteochondrogenic markers were occasionally observed in type III lesions, however, the change did not reach the level of significance compared to type II regions (Fig 3E and 3F). Moreover, the amount of calcification corresponded with a significant increase in cMGP, ucMGP, BMP2 and osteocalcin in type IV regions only (Fig 3D–3F).

**Fig 4:**
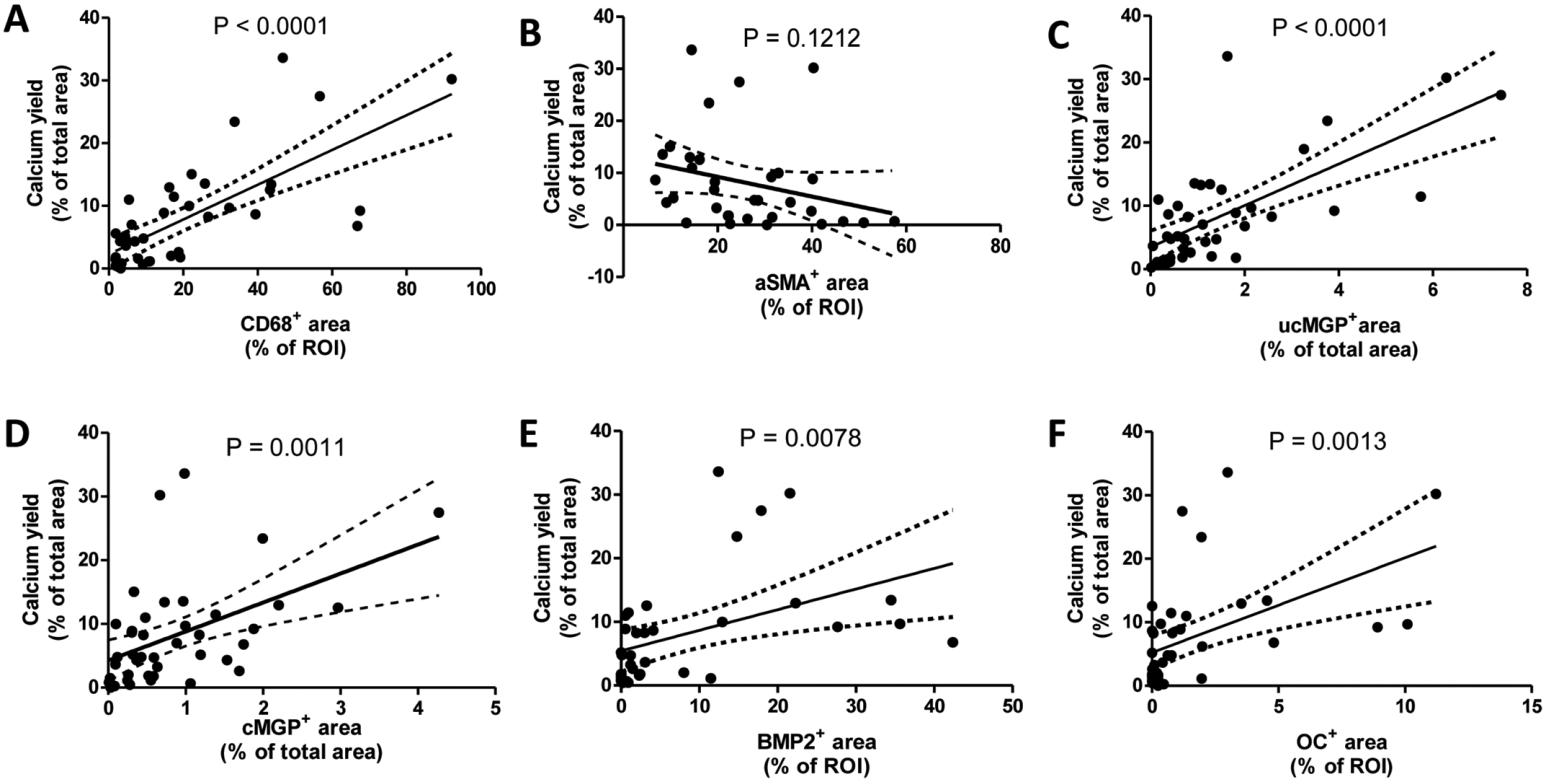
Correlation of amount of micro-calcification with expressions of proteinous catalysts and inhibitors of calcification. Correlations of intimal calcification in atherosclerotic regions and expression of markers aSMA, CD68, ucMGP, cMGP, BMP2 and osteocalcin, irrespective of type of lesion. Calcification was calculated according to “Method one” on the calcium yield scan (S2 File) and represents all values obtained in the type I, II, III, IV regions. Regression analyses between calcification and expression of proteinous catalysts and inhibitors of calcification revealed significant correlations with CD68, ucMGP, cMGP, BMP2 and osteocalcin. Values (r^2^) for determining the degree of correlation between calcification and CD68 or ucMGP, was found to be 0.52 and 0.48, respectively. The degree of correlation between calcification and cMGP, BMP2 or osteocalcin was found to be substantially lower, i.e., 0.24, 0.20 and 0.24, respectively. Significance was determined using the Pearson correlation test.

Correlational studies between calcification and immuno-histochemical markers, irrespective of the type of lesion, revealed that calcification significantly correlated with CD68 positive cells, cMGP, ucMGP, BMP2 and osteocalcin (Fig 4A–4F). The best fit was between calcification and CD68 and ucMGP (r^2^ values of 0.52 and 0.48, respectively). Less correlation was found between calcification and cMGP, BMP2 and osteocalcin (r^2^ values of 0.24, 0.20 and 0.24, respectively). Additionally, the increase in calcification correlated with a non-significant decrease in VSMC content as depicted in Fig 4B (P = 0.12).

### SMC calcification triggers macrophage migration

**Fig 5:**
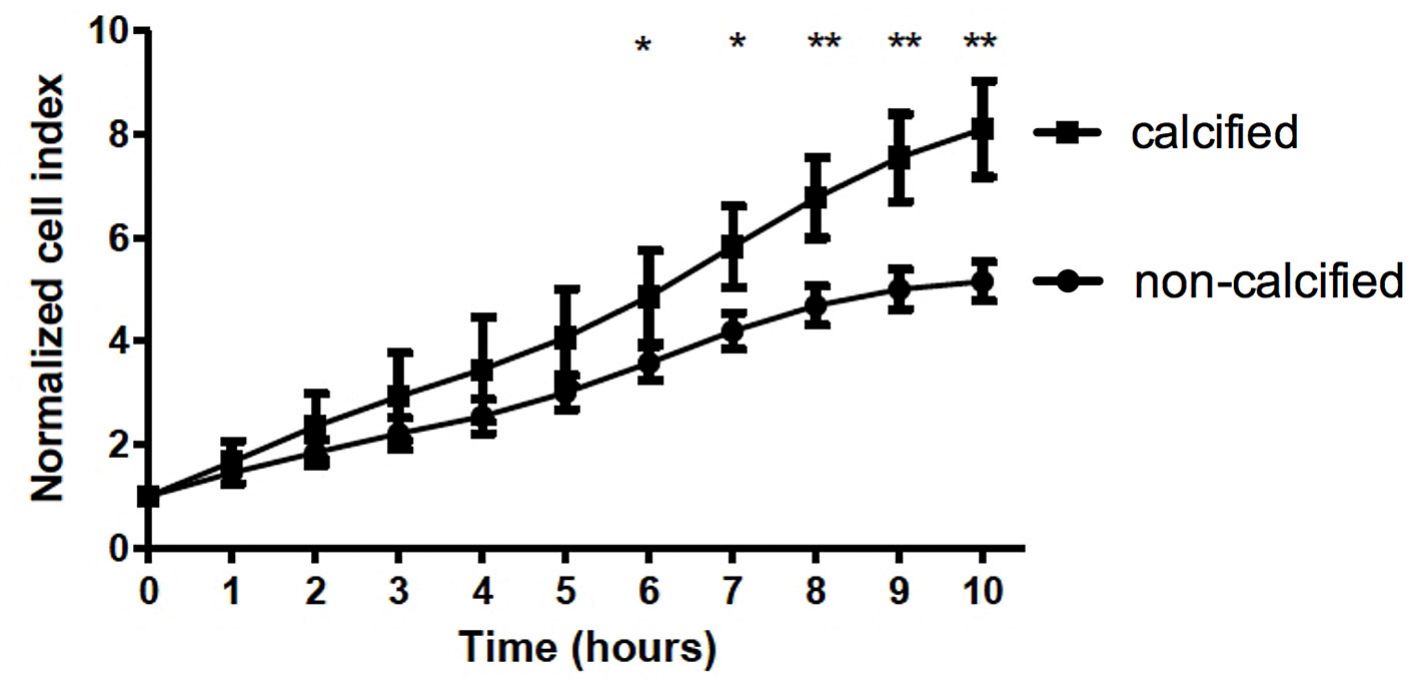
*In vitro* model for chemoattractant properties of calcifying VSMCs on macrophages. The effect of calcifying VSMCs on the attraction of inflammatory cells was tested via invasion assays using PMA-stimulated THP-1 cells (macrophages). Conditioned medium of both control and calcifying VSMCs was used and calcium was added to control medium to obtain equal concentration of calcium in both conditions. Medium from calcifying VSMCs increased the invasion of macrophages significantly, indicating that VSMCs that calcify produce chemoattractants for inflammatory cells. Results were normalised to cell number at start. *P < 0.05, **P < 0.001 significance was assessed unpaired non-parametric t-test (Mann-Whitney).

Since micro-calcifications significantly correlated with number of CD68 positive cells (Fig 3A) we explored the mutual relationship between calcium crystal formation and activation of inflammatory cells in an *in vitro* set up. Interestingly, significant number of macrophages was often observed in the vicinity of calcified areas. We hypothesized that calcifying VSMCs secrete potential chemo-attractants thereby attracting inflammatory cells. To study the effect of calcification on inflammation, we used an *in vitro* xCELLigence based Boyden chamber model to study chemo-attractant properties of VSMCs calcification. VSMCs were grown until confluence, after which half of the VSMCs were put on calcifying medium (2.5% FCS, 5.4 mM CaCl_2_) and the other half on normal medium (2.5% FCS, 1.8 mM CaCl_2_). Medium was harvested after 2 days, and calcium levels were measured and adjusted to the same concentration. Medium from calcifying and non-calcifying VSMCs was used in the lower chamber. Medium from calcified VSMCs displayed a significant increase in THP-1 migration compared to medium of non-calcified VSMCs (Fig 5).

**Fig 6:**
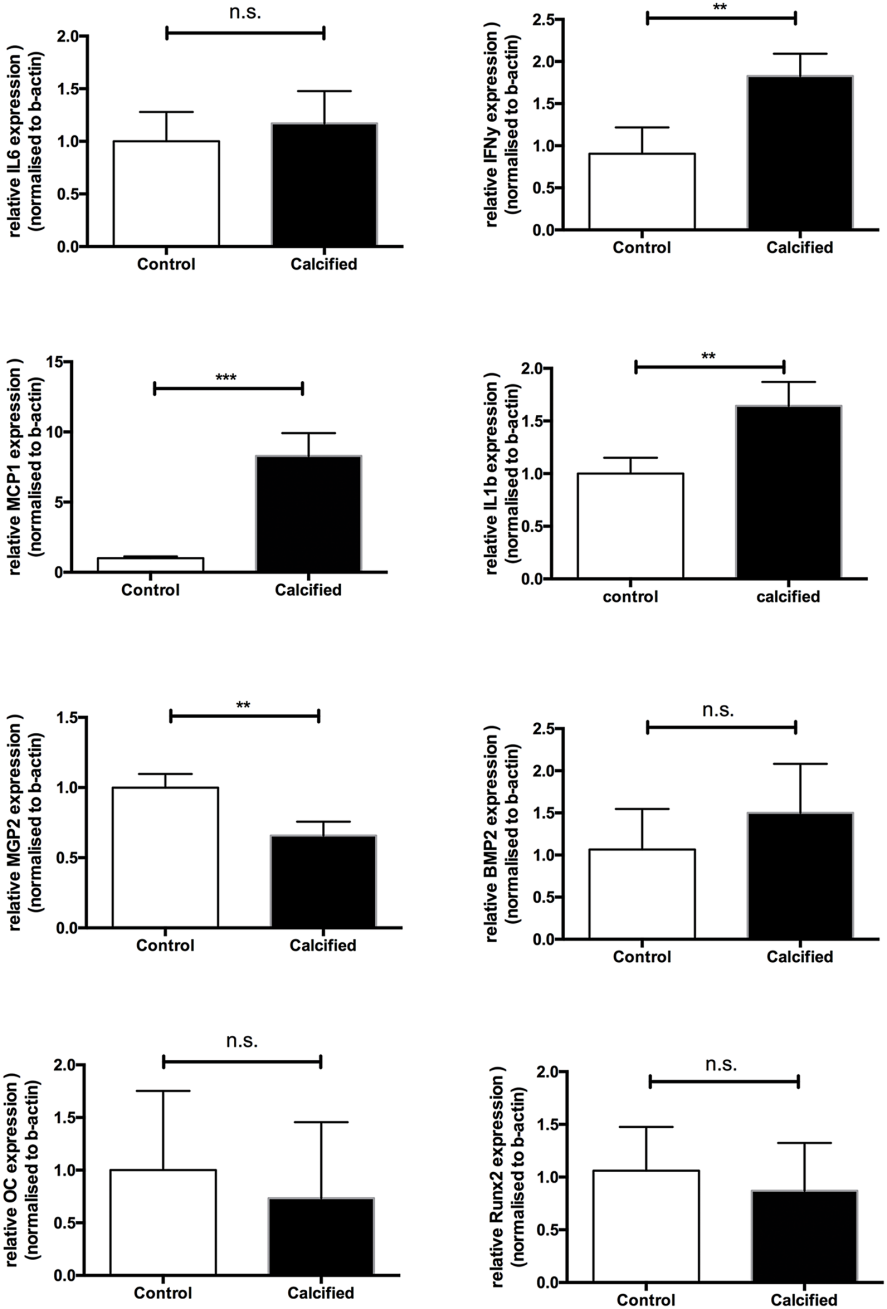
Calcifying VSMCs *in Vitro* display a pro-inflammatory and not an osteochondrogenic phenotype. qPCR of calcifying human primary VSMCs show a significant decrease in MGP as compared to control VSMCs. No differences were found in the expression of the osteochondrogenic markers Runx2, BMP-2 and osteocalcin. The pro-inflammatory cytokines MCP1, IL1b and IFNy were significantly increased in calcifying VSMCs indicative that calcifying VSMCs can initiate local vascular inflammation and promote macrophage migration towards the vascular wall.

Next, we measured in control and calcified VSMCs expression levels of proteins involved in calcification pathways including osteochondrogenesis (MGP, BPM-2, Runx2, osteocalcin). qPCR revealed a significantly lower expression of MGP mRNA in calcifying VSMCs than in control VSMCs (Fig 6). No difference between control and calcified VSMCs was found for BMP-2, Runx2 and osteocalcin, indicating that VSMCs calcify without the need for osteochondrogenic transdifferentiation. Additionally, we analysed expression levels of cytokines known to affect macrophage migration, including IL6, IFNy, MCP1 and IL1b. MCP1, IL1b and IFNy were significantly increased in calcifying VSMCs compared to control, indicating a pro-inflammatory milieu induced by calcified VSMCs.

## Discussion

**Fig 7:**
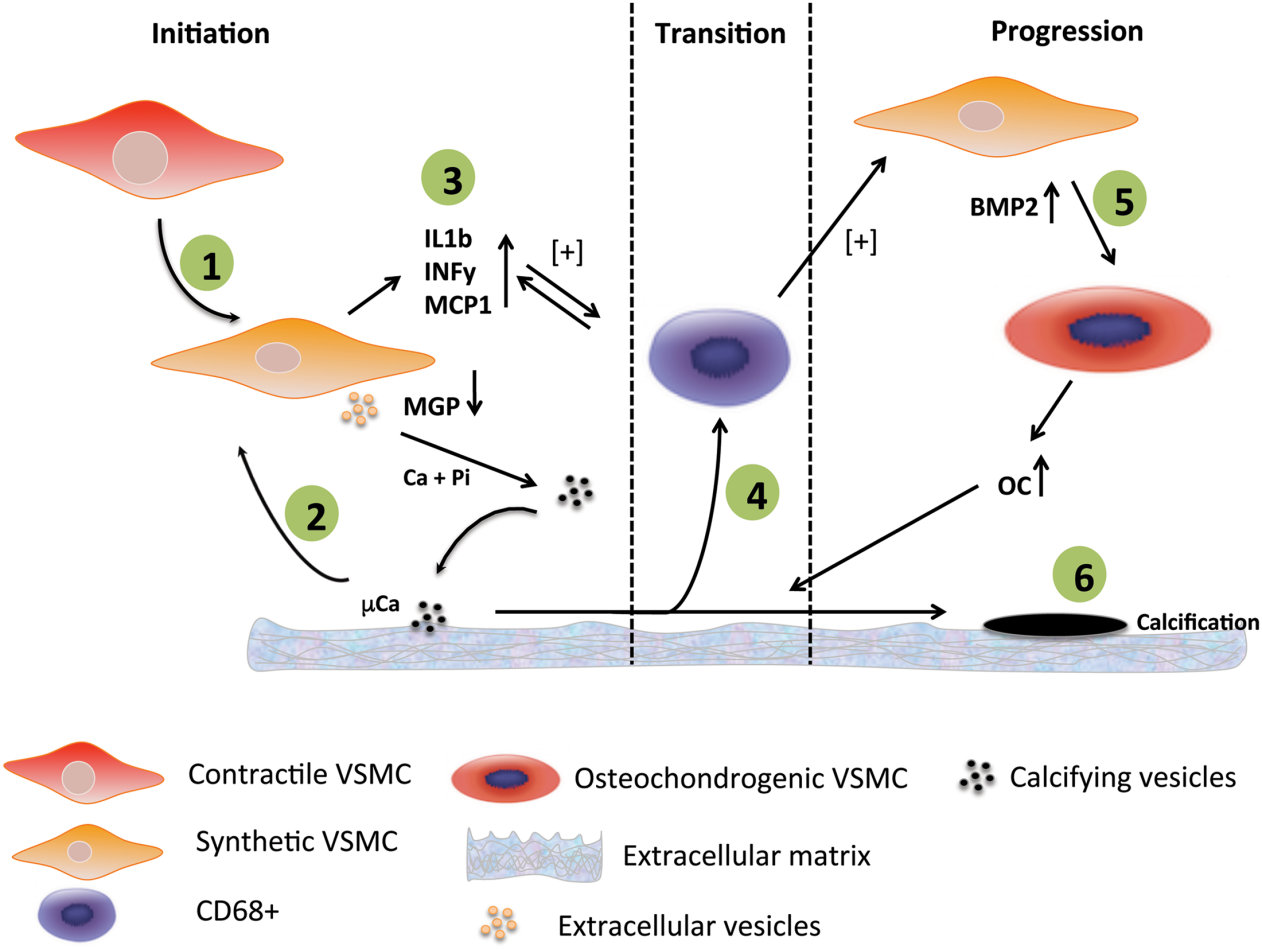
Model showing the potential mechanism of initiation and progression of calcification of the vascular wall. 1) Contractile VSMCs in the thickened intima change phenotype towards synthetic VSMCs. Synthetic VSMCs start secreting extracellular vesicles into the extracellular environment. In case of shortage of vitamin K, a vitamin required for the conversion of ucMGP into the active form cMGP, extracellular vesicles are loaded with ucMGP which is unable to prevent nucleation of calcium-phosphate. 2) Calcifying vesicles provide the first nidus for mineralisation and microcalcifications will be formed. These microcalcifications induce an inflammatory response in VSMCs. 3) VSMCs start secreting pro-inflammatory cytokines that will attract macrophages. 4) Macrophages start fueling the inflammation process by phagocytosing mcirocalcifications and secreting pro-inflammatory cytokines. 5) Pro-inflammatory macrophages affect synthetic VSMCs which will in turn produce BMP2. Synthetic VSMCs will transdifferentiate towards osteochondrogenic VSMCs that subsequently will produce bone-forming proteins such as osteocalcin. 6) Macrocalcifications are the final result of the osteochondrogenic environment in the atherosclerotic plaque.

In the present study we demonstrate that calcification at the micrometer scale is one of the earliest events in the genesis of atherosclerosis. First signs of calcifications are noticed along the elastica interna. The strong correlation with ucMGP suggests that loss of inhibitors and/or a defective inhibitory capacity is causative for the initiation of atherosclerosis. Our data indicate that calcification of VSMCs results in the release of chemoattractants that drive macrophage infiltration, thereby accelerating the propensity for calcification and atherosclerosis. This concept is shown in Fig 7. Finally, only in end-stage atherosclerosis, bone markers were present suggesting that osteochondrogenic differentiation of VSMCs is a consequence of atherosclerotic calcification, rather than being causative.

There is little consensus on how intimal calcification is initiated. In recent years, many initiation routes have been hypothesized [10]. One research line fosters the idea that VSMCs need to acquire an osteogenic profile before calcification occurs [15]. In this view, osteochondrogenic VSMCs produce bone related proteins, such as BMP-2 and osteocalcin, thereby promoting vascular calcification. Secondly, apoptosis of VSMCs has also been put forward as initiation pathway leading to vascular calcification [16,17]. An increased amount of apoptotic bodies and cellular debris serves as nidus for calcification in the extracellular environment. A third concept is the loss of inhibitors, such as MGP, fetuin-A and osteopontin, which normally prevent deposition of calcium crystals in the vascular wall [18]. Loss of inhibitors shifts the balance and allows the nucleation of calcium-phosphate that will eventually lead to the formation of micro-calcifications.

### Initiation of vascular calcification

In this study we followed an alternative immuno-histochemical approach, based on regional heterogeneity, to investigate the sequence of events leading to the genesis of atherosclerotic calcification in human coronary arteries. Atherosclerosis is known to develop with aging and starting with intimal thickening and proliferation of VSMCs is a key event in the genesis of atherosclerosis [19]. These synthetic VSMCs are key in the initiation of vascular calcification via the release of exosomes [20]. We used elemental analysis using a state of the art 3-MeV proton beam which enabled us to quantify micro-calcifications in early stages of atherosclerosis. These first minute depositions of calcium crystals would normally not be detected by conventional calcification stains such as von Kossa or alizarin red. Micro-calcification correlated with increased staining of uncarboxylated MGP (ucMGP), but not of carboxylated MGP (cMGP), analyzed in adjacent sections in selected regions. Carboxylated MGP (cMGP) is known to be one of the key inhibitors of vascular calcification [21]. The conclusion can be drawn that the present data support the notion that calcification starts with the imbalance between inhibitors (relative shortage of cMGP) and activators (e.g formation of extracellular vesicles as nidus for calcification [20], as shown in Fig 7. Several groups demonstrated that MGP expression levels are upregulated in response to calcification [22–25]. Our study confirms accumulation of MGP in areas of calcification, however predominantly in the uncarboxylated conformation that is associated with micro-calcifications along the internal elastic lamina, which is in line with previous findings [26]. Here, we hypothesize that increased levels of ucMGP due to low vitamin K tissue stores result in failure to inhibit the increase in calcium crystals deposition. This corroborates with findings that vitamin K-antagonists induce vitamin K-deficiency and subsequently increase vascular calcification [27].

In our study on human coronary vessel walls, we were unable to detect osteochondrogenic markers in early stages of atherosclerosis, suggesting that osteochondrogenesis is a consequence rather than an initiator of intimal calcification. Using cultured VSMCs we confirmed that calcification can take place without the need of osteochondrogenic differentiation. These *in vitro* findings also showed decreased expression levels of MGP in calcifying VSMCs. The latter data are in line with previous work showing that MGP is first upregulated in VSMCs in response to high levels of extracellular calcium but significantly decreases after 48 hours of incubation [28]. Additionally, we did observe a marked increase of pro-inflammatory cytokines in calcifying VSMCs indicating that calcified VSMCs can act as initiators of local vascular inflammation. We consider the inflammatory response the transition from the initial phase towards the progression phase of vascular calcification (Fig 7).

### Progression of calcification

Since advanced atherosclerotic areas, belonging to type III and IV, also increased amounts of cMGP were observed, the question could be posed why the increase of this inhibitor is not capable of blocking further increase in calcium deposition [29,30]. Most likely, the calcification pressure that is accelerated by CD68 positive cells outbalances the inhibitory capacity of MGP. It is also known that osteochondrogenesis leads to increased secretion of calcification prone vesicles [31]. These data together suggest that in late atherosclerotic stages the osteogenic environment fuels the calcification (Fig 7).

Atherosclerosis is a chronic inflammatory disease characterized by infiltration of inflammatory cells at an early stage [32,33]. In type III lesions, the presence of an a-cellular core with surrounding collagen-rich matrix correlated with micro-calcification, suggesting that VSMCs are able to initiate the calcification process. This is in accordance with previous work showing that collagen promotes calcification of VSMCs [34]. These authors found less macrophage infiltration in atherosclerotic plaques of Ddr1-/- mice that have decreased calcification. Our findings are in line with their observations as we demonstrated that calcifying VSMCs promote migration of macrophages *in vitro*. In our study we noticed a positive correlation between the number of CD68+ cells and amount of calcification. *In vitro* studies showed that calcium crystals promote a pro-inflammatory response in macrophages [7] pointing towards a feed-forward loop (Fig 7). More specifically, macrophages phagocytizing calcium crystals secreted IL-1β and IL-18 through activation of the NLRP-3 inflammasome [35]. We found increased staining of the osteochondrogenic markers BMP-2 and osteocalcinin regions with high number of CD68+ cells, predominantly in type III and IV regions. Previous studies showed that macrophages promote trans-differentiation of VSMC by inducing osteogenic signals [36] and promote VSMC proliferation and migration [37]. Furthermore, BMP signalling was found to exert key regulatory effects in vascular disease [30]. Our data support these findings, as CD68+ cell presence preceded BMP-2 and osteocalcin expression. The positive correlation between the number of CD68+ cells and micro-calcification can also be explained by increased phagocytic scavenging of dying VSMCs. *In vitro* studies showed that calcium crystals promote VSMC apoptosis [8], which induces inflammation [38] and subsequently accelerates atherosclerosis and promotes calcification [17].

## Conclusion

In conclusion, we showed that small punctate calcifications are present at the early stage of atherosclerosis, in association with a deficient inhibitory capacity of mineralisation-regulating proteins, such as cMGP. Vascular calcification serves as chemoattractive signal for macrophages, fuelling the inflammation driven osteochondrogenic VSMC switching. Our findings therefore imply that early-stage micro-calcifications might play a key role in the genesis and progression of cardiovascular disease, and form a target to combat atherosclerotic disease.

## Supporting Information

S1 File Analyses of calcium yield scan. For quantification of the area occupied by micro-calcifications in the calcium yield scans, the media in the image was cropped leaving an image with intimal tissue only. Then, the calcium yield scans were converted to grey values ranging from 0–255. The colours in the original calcium yield scans ranged from light blue—intense blue—red and yellow. In the colour images (Figure A), intensively calcified spots, depicted in red and yellow, were translated to grey values <100. The first signs of calcification (intense blue) were translated to values ranging from 100 to 190 (Figures B and C). Values higher than 190 were regarded as background. The total amount of pixels with a value below 190 (Figure C) were calculated and normalized to the total amount of pixels covered by the intimal tissue in de selected region (Figure D), yielding a value for calcification expressed as percentage of tissue.
(TIF)

S2 File Detailed description of immuno-histochemical analyses according to method one of atherosclerotic regions. Adjacent sections were stained with immuno-histochemical markers for calcification (von Kossa), ucMGP and cMGP,. Quantification of (immuno)-histochemical stained regions was performed by isolation of positive staining from the original image and normalized according to tissue area (“Method one”). Calcification was determined according to “Method one” since no nuclei were visible in the calcium yield scans. MGP positivity was calculated according to “Method one”, since MGP is often found in non-cellular regions due to MGP-binding to calcium-phosphate crystals [21]. All regions were quantified in ImageJ. Firstly, the intima was cropped from the original image (Figure B). For “Method one”, the colours Red and Blue were isolated via the colour deconvolution tool in ImageJ yielding an image for red staining (depicting the alkaline phosphatase substrate deposition as shown in Figure C) and blue staining (nuclei stained by Hematoxyline). Separation of colours by the colour deconvolution tool resulted in imperceptible coloration of background pixels (false positive pixels). In order to get rid of any background coloration, a value of 40 was subtracted from the image leaving an image as seen in Figure D. The value of 40 was validated by investigation of multiple sections with different levels of staining. After subtraction of background (Figure D), the image was converted to a black and white image (Figure E). For normalization by intimal tissue, all intimal tissue in the selected region was also converted black and white image (Figure F). The amount of black pixels in Figure E ((immuno-)- histochemical positivity) was divided by the amount of black pixels in Figure F (intimal tissue) and multiplied by 100 resulting in a percentage of positive staining normalized by total intimal tissue in the selected region.
(TIF)

S3 File Detailed description of immuno-histochemical analyses according to method two of atherosclerotic regions. Adjacent sections were stained with immuno-histochemical markers for VSMCs (a-SMA), macrophages (CD68), BMP2 and osteocalcin. Quantification of (immuno)-histochemical stained regions with cell specific staining according to cell nuclei (“Method two”). All regions were quantified in ImageJ. For quantification according to “Method two” the first 4 steps (Figures A-D) are similar to those of “Method one”. For “Method two” the blue image that is created by the colour deconvolution tool is used for isolation of cell nuclei (Figure B). From this blue image a mask is made for the nuclei. Using the nuclei mask, ROIs (with a line width of 4 pixels) were drawn around the nuclei to cover the cell cytoplasm (Figure C). The sum of total pixels covered by the ROIs is later used as a normalization factor. For validation purposes, an overlay was made of the ROIs, on the original image (Figure D). Hereafter, the red image, corresponding with the positive staining of either CD68, BMP2, osteocalcin, or αSMA, is processed similarly as in “Method one” which yielded a picture as in Figure E). Now an overlay is made of Figs B and F, and when the ROI overlaps a region which is red in Figure E, the ROI pixels are given a red colour (Fig F). The total sum of ROI pixels which are red are calculated and divided by the total amount of pixels covered by the ROIs (normalization factor). This number is multiplied by 100, which resulted in a percentage of cellular staining normalized by the total area covered by the ROIs. This number is related to the amount of positive stained cells where the normalization factor corresponds to amount of cells found in the selected region.
(TIF)
