# Supplementary figures and images for: Intra-Section Analysis of Human Coronary Arteries Reveals a Potential Role for Micro-Calcifications in Macrophage Recruitment in the Early Stage of Atherosclerosis
Source: PLoS One. 2015 Nov 10;10(11):e0142335. doi: 10.1371/journal.pone.0142335 (PMC4640818; doi:10.1371/journal.pone.0142335)

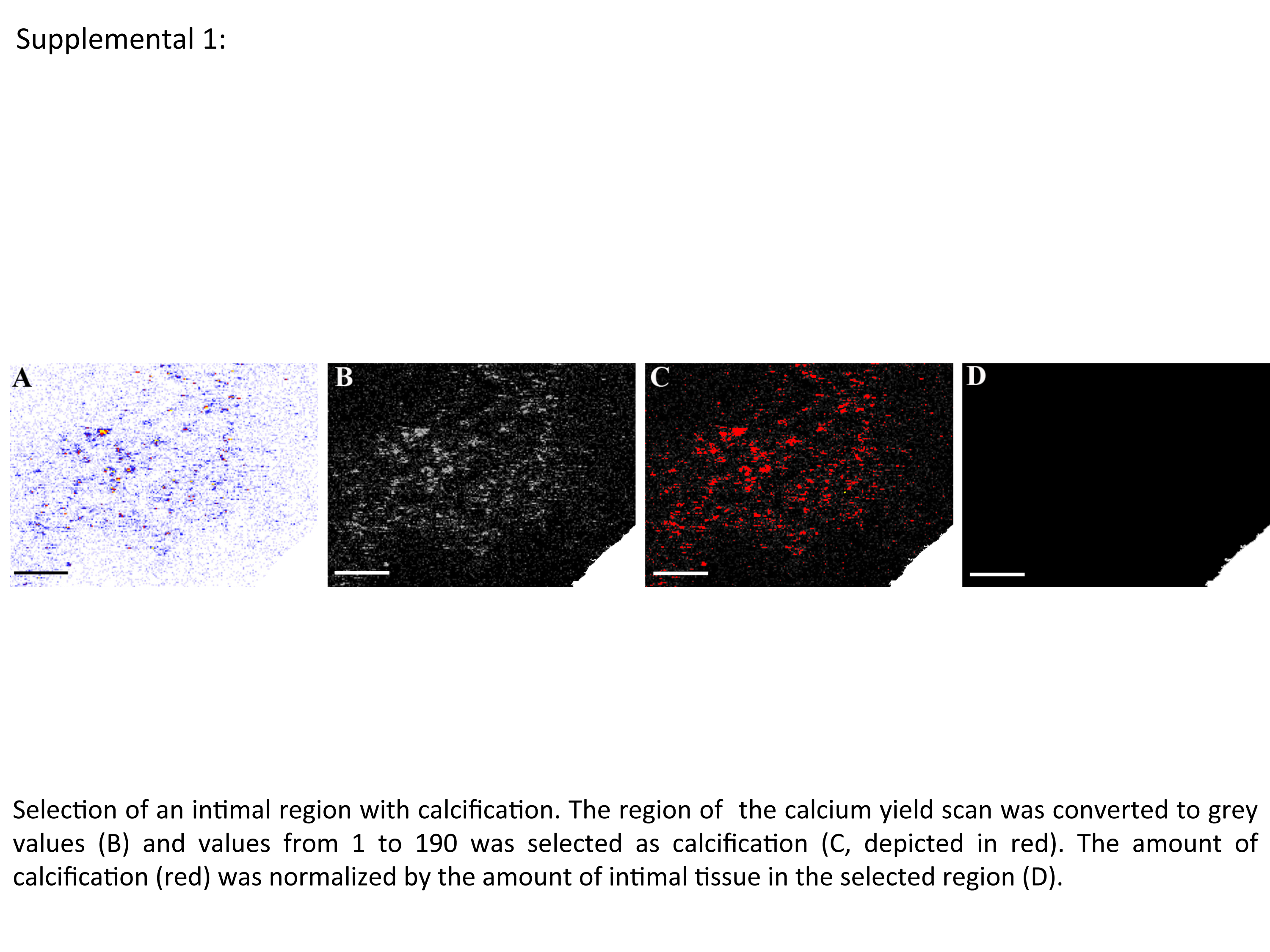

Supplement: S1 File — For quantification of the area occupied by micro-calcifications in the calcium yield scans, the media in the image was cropped leaving an image with intimal tissue only. Then, the calcium yield scans were converted to grey values ranging from 0–255. The colours in the original calcium yield scans ranged from light blue—intense blue—red and yellow. In the colour images (Figure A), intensively calcified spots, depicted in red and yellow, were translated to grey values <100. The first signs of calcification (intense blue) were translated to values ranging from 100 to 190 (Figures B and C). Values higher than 190 were regarded as background. The total amount of pixels with a value below 190 (Figure C) were calculated and normalized to the total amount of pixels covered by the intimal tissue in de selected region (Figure D), yielding a value for calcification expressed as percentage of tissue. (TIF) [file pone.0142335.s001.tif]

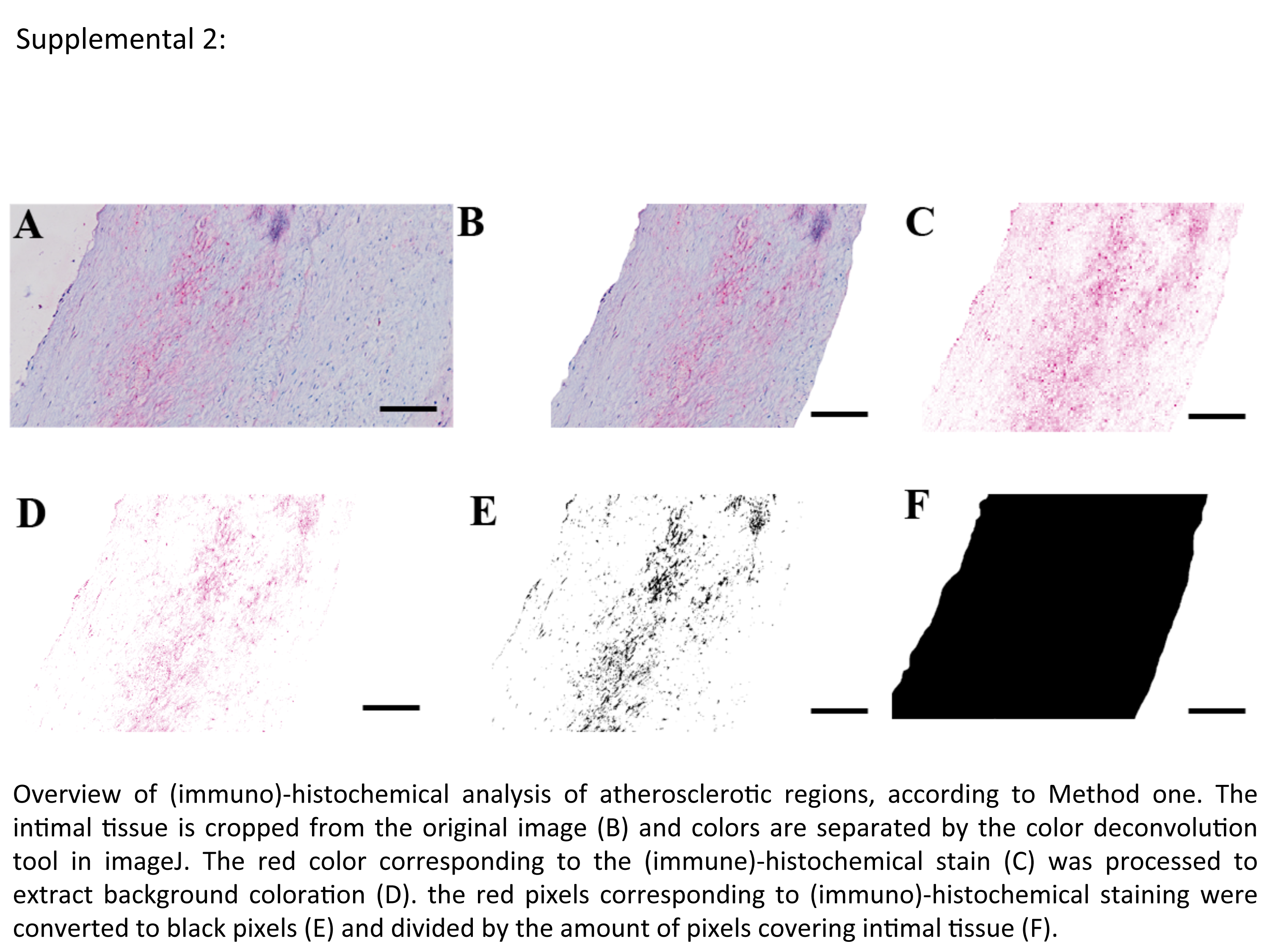

Supplement: S2 File — Adjacent sections were stained with immuno-histochemical markers for calcification (von Kossa), ucMGP and cMGP,. Quantification of (immuno)-histochemical stained regions was performed by isolation of positive staining from the original image and normalized according to tissue area (“Method one”). Calcification was determined according to “Method one” since no nuclei were visible in the calcium yield scans. MGP positivity was calculated according to “Method one”, since MGP is often found in non-cellular regions due to MGP-binding to calcium-phosphate crystals [21]. All regions were quantified in ImageJ. Firstly, the intima was cropped from the original image (Figure B). For “Method one”, the colours Red and Blue were isolated via the colour deconvolution tool in ImageJ yielding an image for red staining (depicting the alkaline phosphatase substrate deposition as shown in Figure C) and blue staining (nuclei stained by Hematoxyline). Separation of colours by the colour deconvolution tool resulted in imperceptible coloration of background pixels (false positive pixels). In order to get rid of any background coloration, a value of 40 was subtracted from the image leaving an image as seen in Figure D. The value of 40 was validated by investigation of multiple sections with different levels of staining. After subtraction of background (Figure D), the image was converted to a black and white image (Figure E). For normalization by intimal tissue, all intimal tissue in the selected region was also converted black and white image (Figure F). The amount of black pixels in Figure E ((immuno-)- histochemical positivity) was divided by the amount of black pixels in Figure F (intimal tissue) and multiplied by 100 resulting in a percentage of positive staining normalized by total intimal tissue in the selected region. (TIF) [file pone.0142335.s002.tif]

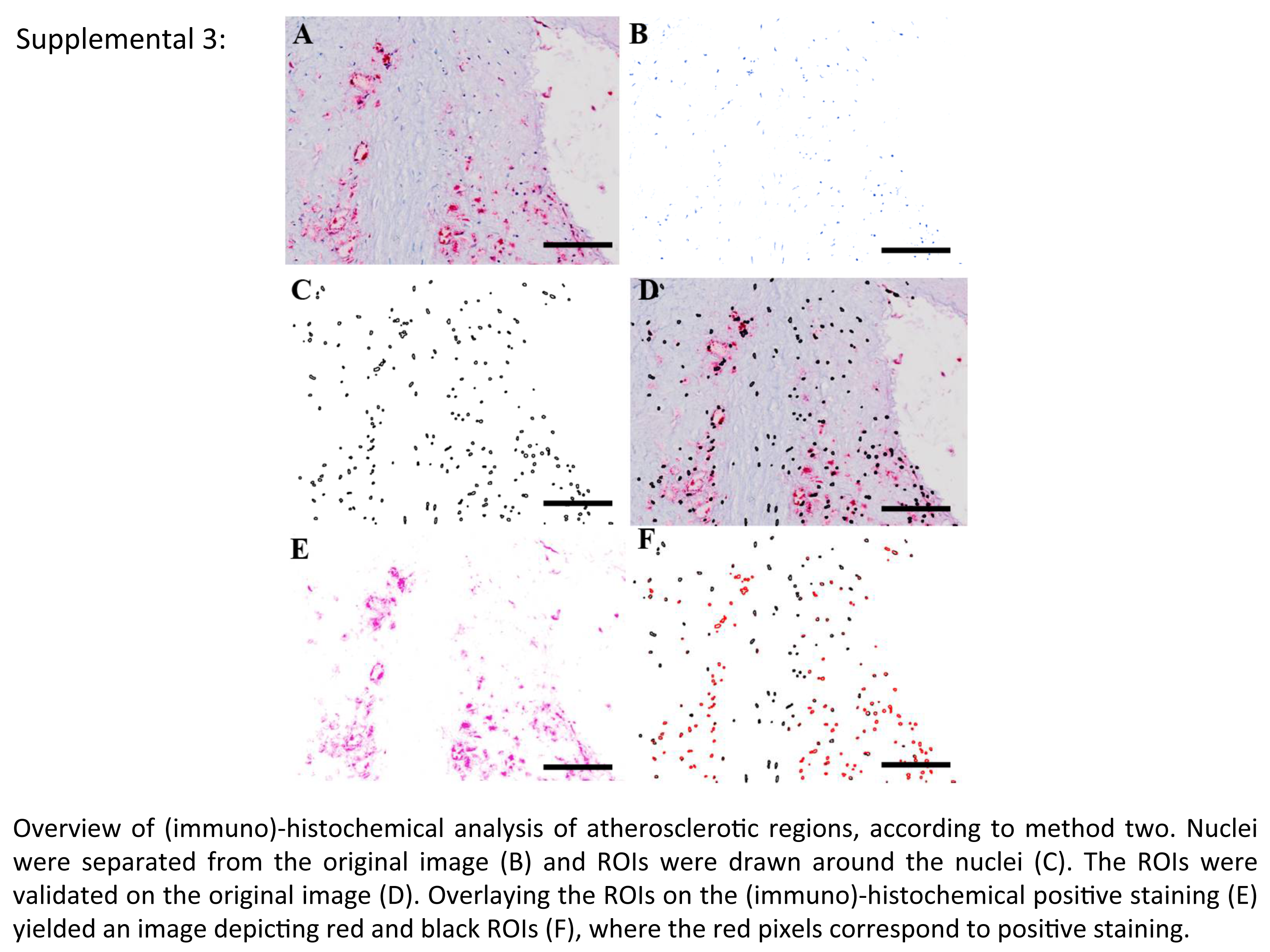

Supplement: S3 File — Adjacent sections were stained with immuno-histochemical markers for VSMCs (a-SMA), macrophages (CD68), BMP2 and osteocalcin. Quantification of (immuno)-histochemical stained regions with cell specific staining according to cell nuclei (“Method two”). All regions were quantified in ImageJ. For quantification according to “Method two” the first 4 steps (Figures A-D) are similar to those of “Method one”. For “Method two” the blue image that is created by the colour deconvolution tool is used for isolation of cell nuclei (Figure B). From this blue image a mask is made for the nuclei. Using the nuclei mask, ROIs (with a line width of 4 pixels) were drawn around the nuclei to cover the cell cytoplasm (Figure C). The sum of total pixels covered by the ROIs is later used as a normalization factor. For validation purposes, an overlay was made of the ROIs, on the original image (Figure D). Hereafter, the red image, corresponding with the positive staining of either CD68, BMP2, osteocalcin, or αSMA, is processed similarly as in “Method one” which yielded a picture as in Figure E). Now an overlay is made of Figs B and F, and when the ROI overlaps a region which is red in Figure E, the ROI pixels are given a red colour (Fig F). The total sum of ROI pixels which are red are calculated and divided by the total amount of pixels covered by the ROIs (normalization factor). This number is multiplied by 100, which resulted in a percentage of cellular staining normalized by the total area covered by the ROIs. This number is related to the amount of positive stained cells where the normalization factor corresponds to amount of cells found in the selected region. (TIF) [file pone.0142335.s003.tif]
